# Rapid and Concomitant Gut Microbiota and Endocannabinoidome Response to Diet-Induced Obesity in Mice

**DOI:** 10.1128/mSystems.00407-19

**Published:** 2019-12-17

**Authors:** Sébastien Lacroix, Florent Pechereau, Nadine Leblanc, Besma Boubertakh, Alain Houde, Cyril Martin, Nicolas Flamand, Cristoforo Silvestri, Frédéric Raymond, Vincenzo Di Marzo, Alain Veilleux

**Affiliations:** a Institut sur la nutrition et les aliments fonctionnels (INAF), Québec, Québec, Canada; b Centre de recherche de l’Institut universitaire de cardiologie et de pneumologie de Québec (IUCPQ), Québec, Québec, Canada; c École de nutrition, Faculté des sciences de l’agriculture et de l’alimentation (FSAA), Université Laval, Québec, Québec, Canada; d Département de médecine, Faculté de Médecine, Université Laval, Québec, Québec, Canada; e Joint International Unit between the National Research Council (CNR) of Italy and Université Laval on Chemical and Biomolecular Research on the Microbiome and its Impact on Metabolic Health and Nutrition (UMI-MicroMeNu), Institute of Biomolecular Chemistry, CNR, Pozzuoli, Italy; f Canada Excellence Research Chair on the Microbiome-Endocannabinoidome Axis in Metabolic Health (CERC-MEND), Université Laval, Québec, Québec, Canada; Duke University

**Keywords:** obesity, gut microbiota, endocannabinoidome, intestine, high-fat, high-sucrose

## Abstract

The intestinal microbiota and the expanded endocannabinoid (eCB) system, or endocannabinoidome (eCBome), have both been implicated in diet-induced obesity and dysmetabolism. These systems were recently suggested to interact during the development of obesity. We aimed at identifying the potential interactions between gut microbiota composition and the eCBome during the establishment of diet-induced obesity and metabolic complications. Male mice were fed a high-fat, high-sucrose (HFHS) diet for 56 days to assess jejunum, ileum, and cecum microbiomes by 16S rRNA gene metataxonomics as well as ileum and plasma eCBome by targeted liquid chromatography-tandem mass spectrometry (LC-MS/MS). The HFHS diet induced early (3 days) and persistent glucose intolerance followed by weight gain and hyperinsulinemia. Concomitantly, it induced the elevation of the two eCBs, anandamide, in both ileum and plasma, and 2-arachidonoyl-glycerol, in plasma, as well as alterations in several other *N*-acylethanolamines and 2-acylglycerols. It also promoted segment-specific changes in the relative abundance of several genera in intestinal microbiota, some of which were observed as early as 3 days following HFHS diet. Weight-independent correlations were found between the relative abundances of, among others, *Barnesiella*, *Eubacterium*, *Adlercreutzia*, *Parasutterella*, *Propionibacterium*, *Enterococcus*, and *Methylobacterium* and the concentrations of anandamide and the anti-inflammatory eCBome mediator *N*-docosahexaenoyl-ethanolamine. This study highlights for the first time the existence of potential interactions between the eCBome, an endogenous system of multifunctional signaling lipids, and several intestinal genera during early and late HFHS-induced dysmetabolic events, with potential impact on the host capability of adapting to increased intake of fat and sucrose.

**IMPORTANCE** The intestinal microbiota and the expanded endocannabinoid system, or endocannabinoidome, have both been implicated in diet-induced obesity and dysmetabolism. This study aims at identifying the potential interactions between these two fundamental systems—which form the gut microbiota-endocannabinoidome axis—and their involvement in the establishment of diet-induced obesity and related metabolic complications. We report here time- and segment-specific microbiome disturbances as well as modifications of intestinal and circulating endocannabinoidome mediators during high-fat, high-sucrose diet-induced glucose intolerance and subsequent obesity and hyperinsulinemia. This highlights the involvement of, and the interaction between, the gut microbiota and the endocannabinoidome during metabolic adaptation to high-fat and high-sucrose feeding. These results will help identifying actionable gut microbiome members and/or endocannabinoidome mediators to improve metabolic health.

## Abstract (precis)

The intestinal microbiota and the expanded endocannabinoid system, or endocannabinoidome, have both been implicated in diet-induced obesity and dysmetabolism. This study aims at identifying the potential interactions between these two fundamental systems—which form the gut microbiota-endocannabinoidome axis—and their involvement in the establishment of diet-induced obesity and related metabolic complications. We report here time- and segment-specific microbiome disturbances as well as modifications of intestinal and circulating endocannabinoidome mediators during high-fat, high-sucrose diet-induced glucose intolerance and subsequent obesity and hyperinsulinemia. This highlights the involvement of, and the interaction between, the gut microbiota and the endocannabinoidome during metabolic adaptation to high-fat and high-sucrose feeding. These results will help identifying actionable gut microbiome members and/or endocannabinoidome mediators to improve metabolic health.

## INTRODUCTION

The current rise in the prevalence of obesity, insulin resistance, and their cardiometabolic comorbidities could in part result from the incompatibility between the microbial species lining the intestinal wall, host metabolism, and modifications of nutritional habits (1). Increased consumption of animal fats or simple sugars can indeed lead to compositional and functional modifications of the gut microbiome, potentially inducing dysbiosis and local/systemic repercussions such as impaired intestinal function, immune response, inflammation, insulin resistance, and dysmetabolism (2, 3).

The consequences of dysbiosis may result from either direct bacterial-intestinal wall interactions or indirect bacterium-host interactions mediated by gut-derived metabolites. One such class of molecules are the endocannabinoids (eCBs), i.e., *N*‑arachidonoyl‑ethanolamine (anandamide [AEA]) and 2-arachidonoyl-glycerol (2-AG), which act at cannabinoid type 1 (CB1) and type 2 (CB2) receptors and have been implicated in the control of energy metabolism and inflammation, among other biological processes (4, 5). In particular, CB1 overactivity is considered a key contributor to the development of obesity and associated metabolic disturbances (6). Moreover, some mediators related to the eCBs—the *N*-acylethanolamines (AEA congeners) and 2-acylglycerols (2-AG congeners)—share biosynthetic and catabolic enzymes with the two eCBs but act on different molecular targets, such as the transient receptor potential vanilloid type 1 (TRPV1) channel, the peroxisome proliferator-activated receptors (PPARs) α and γ and the orphan G-protein-coupled receptors GPR119 and GPR55. Unlike CB1 but like CB2 receptors, activation of these molecular targets has been associated with amelioration of glucose intolerance, perturbed intestinal permeability, insulin resistance, and/or obesity (7–9). Altogether, these biomolecules, their metabolic enzymes, and their various receptors form the endocannabinoidome (eCBome) (10). The complex network of target receptors and metabolic functions of each eCBome mediator is summarized in Table S1 in the supplemental material.

10.1128/mSystems.00407-19.4 TABLE S1 Endocannabinoidome targets and mediators. Table detailing all endocannabinoidome targets studied, their mediators, and their roles in energy metabolism, small intestine functions, and systemic inflammation. Download Table S1, DOCX file, 0.1 MB. Copyright © 2019 Lacroix et al. 2019 Lacroix et al. This content is distributed under the terms of the Creative Commons Attribution 4.0 International license.

Modulation of gut microbiota composition by diet and prebiotics influences the profile of eCBome mediators (11). Conversely, modulation of eCBome signaling, through either anabolic enzyme gene knockout (i.e., in adipocyte or intestinal epithelial cell-specific *Napepld* null mice) (12) or receptor agonists and antagonists (13), was shown to impact the gut microbiome community. Interestingly, CB1 and TRPV1 agonists may produce opposite effects (i.e., negative or positive modulation, respectively) on metabolically beneficial (e.g., Akkermansia muciniphila) commensal microorganisms (11, 14). In either case, the metabolic and systemic inflammatory response to dietary challenges, such as a high-fat diet, are also profoundly and concomitantly altered. Thus, there is evidence of bidirectional interactions between the gut microbiome and the eCBome in the context of metabolic control. Despite these observations, it is not known whether these two systems respond in a coordinated manner to the establishment of diet-induced obesity and related metabolic complications. With most studies focused on the fecal or colon microbiota, the temporal relationship between diet-induced alterations of the gut microbiome and the eCBome is even less well known in the small intestine, which exhibits different anatomical characteristics and microbial composition. In particular, the small intestine, while bearing a lower bacterial load than the colon, plays an important role in nutrient sensing, nutrient absorption, gut hormone secretion, and metabolism (15), all functions in which several eCBome receptors (e.g., CB1, TRPV1, PPARα, and GPR119) have also been deeply implicated, with often opposite effects (4).

With the present study, we have investigated the time-dependent response of the gut microbiota-eCBome axis to a high-fat, high-sucrose (HFHS) diet throughout the small intestine and the cecum. Our results are pivotal in determining the implication of this axis in diet-induced glucose intolerance, obesity, and other metabolic disturbances.

## RESULTS

**FIG 1:**
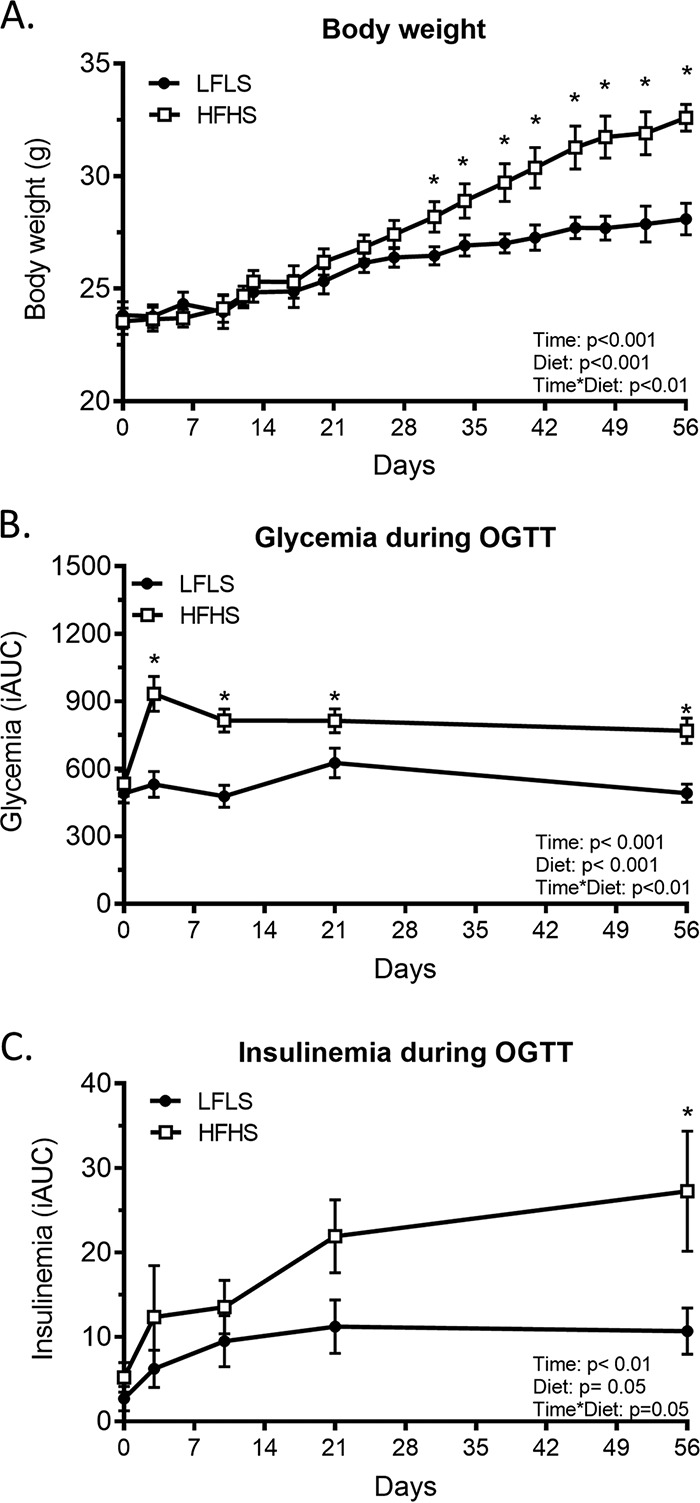
Impact of 56 days of LFLS and HFHS feeding on mouse phenotype. Two groups of 11 mice were fed either an LFLS or an HFHS diet for 56 days. (A) Weight gain; (B) plasma glucose area under the OGTT curve (incremental area under the curve [iAUC]); (C) plasma insulin area under the OGTT curve (iAUC). Mixed linear regression and generalized linear regression models were used to identify time or diet effects and interactions. Data are expressed as mean ± SEM (*n* = 9 to 12). *, *P* < 0.05 for Tukey HSD *post hoc* test between LFLS and HFHS groups.

Mice fed the HFHS diet experienced a higher weight gain than those fed the low-fat, low-sucrose (LFLS) diet, with the two groups significantly diverging from each other from day 31 (32.6 ± 1.8 g versus 28.1 ± 1.6 g at day 56, respectively) (Fig. 1A). Interestingly, the greater weight gain induced by HFHS feeding was preceded by reduction of glucose tolerance as revealed by elevated glucose area under the oral glucose tolerance test (OGTT) curve from day 3 of HFHS feeding (Fig. 1B). Insulin area under the OGTT curves was significantly elevated only at day 56 of HFHS feeding, which suggests progressively decreasing insulin sensitivity over the duration of the protocol accompanying the greater body weight gain (Fig. 1C).

### Segment-specific gut microbiome community reshaping during HFHS diet feeding.

**FIG 2:**
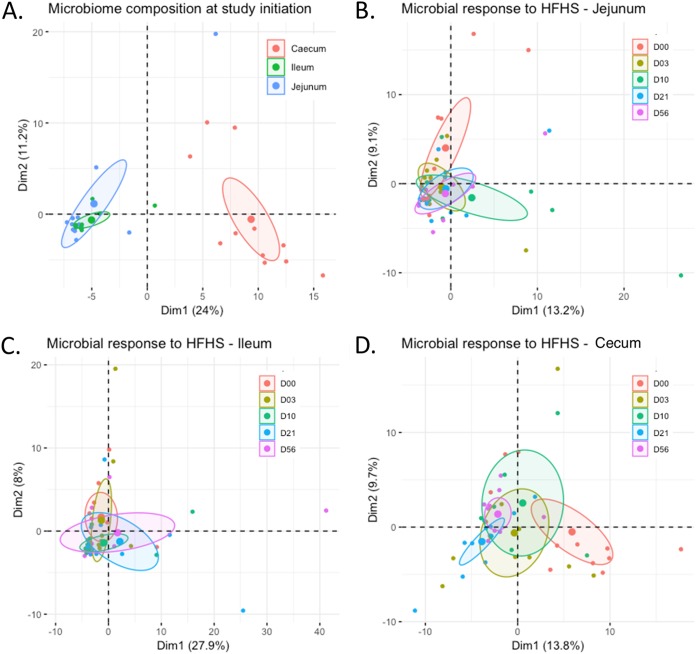
Intestinal microbiota composition in response to the HFHS diet. (A) Principal-component analysis (PCA) of gut microbiota composition in each intestinal segment prior to the initiation of the HFHS diet. (B to D) Impact of HFHS feeding on gut microbiota composition of the jejunum (B), the ileum (C), and the cecum (D). *n* = 6 to 12 per time point.

**FIG 3:**
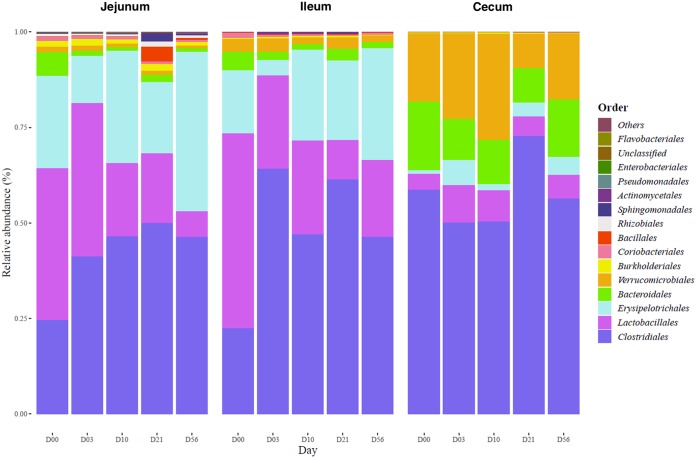
Relative bacterial abundance at the order level in response to the HFHS diet. Orders representing less than 1% of total bacterial abundance in at least one segment were aggregated.

**FIG 4:**
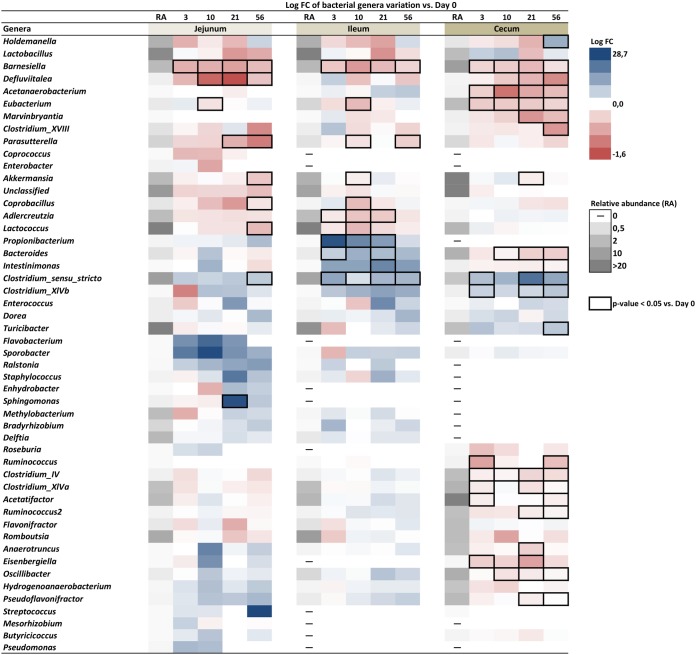
Relative bacterial abundance at the genus level in response to the HFHS diet. Relative bacterial abundances at day 0 are represented in grayscale in the first column of the heatmap of each intestinal segment. Each heatmap illustrates change in relative abundance of each genus expressed as log_2_ fold change (FC) computed against day 0 for each segment. Boxed cells refer to *P* < 0.05 for Kruskal-Wallis test between a specific time point and day 0 (*n* = 6 to 12 per time point).

Principal-component analyses (PCAs) on the gut microbiota composition revealed a clear differentiation between cecum and small intestine segments prior to HFHS diet initiation (Fig. 2A). More specifically, gut microbiota communities corresponded to expectations, with aerobes and facultative anaerobes (e.g., *Bacillales*, *Erysipelotrichales*, and *Lactobacillales*) being favored on average over obligate anaerobes (e.g., *Clostridiales*, *Bacteroidales*, and *Verrucomicrobiales*) in the small intestine segments versus the cecum (Fig. 3). The relative abundance of bacterial genera in each segment is also highlighted in Fig. 4. Bacterial diversity, assessed by the Shannon α-diversity index, was greater in the cecum (3.2 [3.0-3.3]) (values shown as median [Q1-Q3]) than in the jejunum (2.1 [1.8-2.8]) and ileum (2.2 [1.9-2.5], *P* < 0.01), supporting the concept of the existence of a heterogeneous relative abundance of genera in different segments of the small intestine. The *Firmicutes*-to-*Bacteroidetes* ratio was greater in the jejunum (1.46 [1.31-1.65]) and the ileum (1.44 [1.40-1.64]) than in the cecum microbiota (1.22 [1.11-1.24], *P* < 0.01). Based on these observations, the subsequent analyses were performed separately for each intestinal segment to focus on segment-specific responses to the HFHS diet.

HFHS diet feeding did not appear to noticeably impact the global composition of the small intestine microbiota, as all HFHS-fed groups were indistinguishable from the baseline by PCA (Fig. 2B and C). Interestingly, HFHS feeding gradually impacted the composition of the cecum microbiota, which was definitively distinguishable from the baseline at days 21 and 56 (Fig. 2D). Bacterial α-diversity of the cecum microbiota, but not of the other segments, decreased within 3 days of HFHS feeding and returned to baseline by 56 days (*P* < 0.01, data not shown). The *Firmicutes*-to-*Bacteroidetes* ratio remained unchanged in all segments throughout the HFHS feeding period (data not shown), which might reflect the similar fiber contents of the two diets, a marked difference from previous studies that use conventional high-fiber chow diets as controls (16).

Fold changes (FCs) of relative bacterial abundance at the genus level upon initiation of HFHS feeding for each segment are illustrated in Fig. 4. Analysis revealed that relative abundances of *Barnesiella*, *Defluviitalea*, and *Eubacterium* were decreased in both small intestine segments and the cecum as early as 3 or 10 days after the initiation of HFHS feeding. *Akkermansia* also showed a trend for reduced relative abundance in all three segments, which reached significance between 10 and 56 days. *Lactococcus*, *Coprobacillus*, and *Parasutterella* were decreased mostly at later time points in small intestine segments. In contrast, *Clostridium sensu stricto* relative abundance rapidly increased in all segments. Other segment-specific changes were observed in response to HFHS feeding (Fig. 4). In the ileum microbiota, *Propionibacterium* relative abundance rapidly increased at day 3 of HFHS feeding and tended to return to the baseline state at day 56. *Acetanaerobacterium*, *Clostridium* IV, *Clostridium* XVIII, *Eisenbergiella*, *Marvinbryantia*, *Oscillibacter*, and *Ruminococcus* genus abundance was decreased, while *Turicibacter* was increased in the cecum microbiota by HFHS feeding. Interestingly, *Bacteroides* shows segment-specific regulation by HFHS feeding, characterized by a rapid increase in the ileum but a concomitant decrease in the cecum. Taken together, these results highlight both rapid and delayed segment-specific microbiota responses to HFHS feeding in mice, which may be relevant to HFHS-induced glucose intolerance and obesity/insulin resistance, respectively.

### eCBome mediators are modified in response to the HFHS diet.

**FIG 5:**
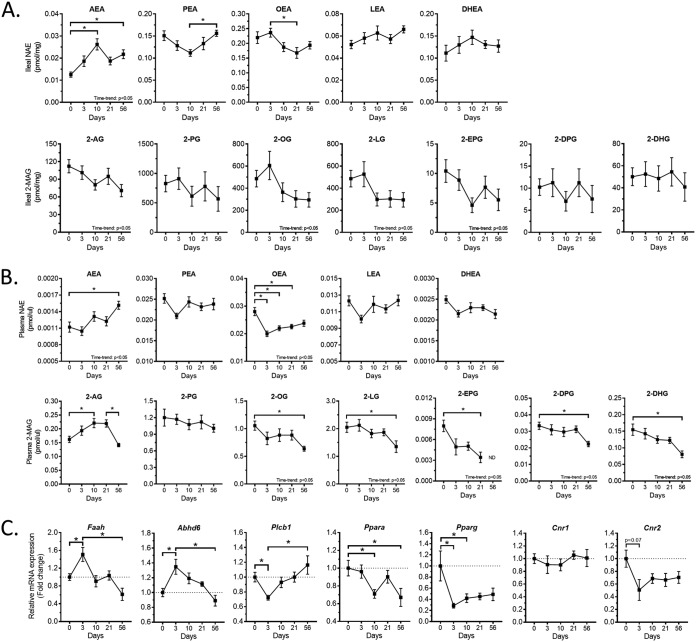
Endocannabinoidome response to the HFHS diet. (A and B) Line chart representation of the endocannabinoidome mediators in the ileum (A) and the plasma (B) at each time point following HFHS feeding initiation. Top row, *N*-acylethanolamines (NAEs). Bottom row, 2-monoacylglycerols (2-MAGs). (C) Ileum mRNA expression of endocannabinoidome-related gene as fold change (FC) calculated using the ΔΔ*C_T_* method. Expression was normalized to *Tbp* and relative to day 0. Data are expressed as the mean ± SEM (*n* = 9 to 12 per time point). *P* values of linear contrast *post hoc* analysis are detailed in the bottom right corners when significant; *, *P* < 0.05 for Tukey HSD *post hoc* test between each time point. ND, not determined.

Through their capability of modulating the activity of molecular targets deeply involved in the control of metabolism, such as CB1 (AEA and 2-AG), PPARα (*N*-oleoylethanolamine [OEA] and *N*-palmitoylethanolamine [PEA]), TRPV1 (all long-chain nonsaturated *N*-acylethanolamines and 2-monoacylglycerols), GPR119 (OEA, *N*-linoleoylethanolamine [LEA], 2-oleoyl-glycerol [2-OG], and 2-linoleoyl-glycerol [2-LG]), and GPR55 (PEA) (10) (see Table S1 in the supplemental material), changes in eCBome mediators have been linked to the development of the metabolic syndrome, obesity, and type 2 diabetes, and their potential bidirectional interplay with gut microbiota has been highlighted (17). We assessed the impact of the HFHS feeding on eCBome mediators in the ileum and the plasma. In the ileum, we observed a significantly positive time trend (analysis of variance [ANOVA] linear contrast *post hoc* analysis) for AEA, which peaked 10 days after HFHS diet initiation (+109% at 10 days, *P* < 0.05). In contrast, the AEA congeners PEA and OEA tended to decrease following 10 days of HFHS feeding, although PEA levels were restored compared to the baseline at day 56 of HFHS feeding. The levels of the anti-inflammatory AEA congener *N*-docosahexaenoylethanolamine (DHEA) did not change with administration of the HFHS diet. The other main eCB, 2-AG, showed a negative time trend although the decrease at day 56 did not reach significance. The 2-AG congeners 2-OG and 2-LG, reported to act as TRPV1 and GPR119 agonists, also showed a significant negative time trend (*P* < 0.05, Fig. 5A).

Important changes in eCBome mediators were also noted in the plasma, where the arachidonic acid-containing eCBs, AEA and 2-AG, were increased (+31% and +50%, respectively; *P* < 0.05) (Fig. 5B). Of note, 2-AG levels in the plasma peaked at days 10 and 21 and then markedly dropped at day 56. Oleoyl (OEA and 2-OG)-, linoleoyl (2-LG)-, and omega-3 [2-EPG, 2-DPG, and 2-DHG]-derived eCBome mediators, however, were mostly decreased by HFHS feeding (*P* < 0.05, Fig. 5B). By design, these changes were achieved in a context where total lipid intake increased by 4.5-fold in the HFHS diet over the LFLS diet, but the composition and the omega-3/omega-6 ratio remained similar.

### Expression of eCBome catabolic enzymes and receptors is modified by HFHS diet.

To explain changes in ileum eCBome mediators, we assessed the impact of the HFHS feeding on mRNA expression of targeted eCBome genes in the ileum. The decreased expression of the AEA-degrading enzyme, *Faah*, following a peak at day 3 corresponded to the increased ileum AEA levels maintained at later time points (Fig. 5C). Similarly, the initial increase of the monoacylglycerol-degrading enzyme *Abhd6* and decrease of the rate-limiting enzyme in 2-monoacylglycerol biosynthesis, *Plcb1*, correlate with the trend decreases of 2-AG, 2-OG, 2-LG, and 2-EPG, although these trends also continued when the expression of the enzymes went back to baseline levels. Finally, we found decreased expression for *Ppara*, *Pparg*, and *Cnr2* (Fig. 5C).

### Interactions between the gut microbiota and the eCBome in response to the HFHS diet.

**FIG 6:**
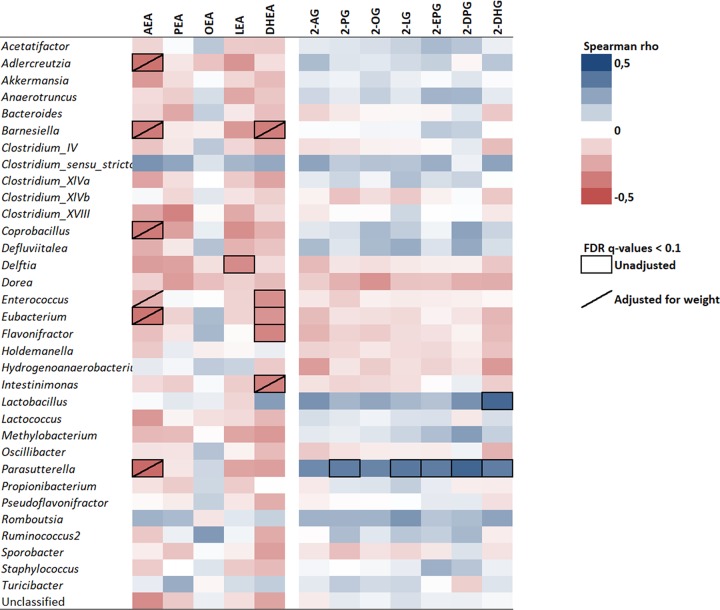
Correlation between ileum microbiota composition and local endocannabinoidome during HFHS feeding. The heatmap illustrates correlation coefficients between the relative abundance of each genus and the level of endocannabinoidome mediators in the ileum. Boxed cells refer to *q* values of <0.1 of FDR-adjusted Spearman rho correlations; slashed cells refer to *q* values of <0.1 of FDR-adjusted partial Spearman rho correlations accounting for weight at sacrifice; *n* = 49 to 58.

Apart from attempting to identify parallel changes with time of both gut microbiota genera and eCBome mediators based on the results described above, we also investigated the interactions between gut microbiota genera and eCBome mediators using Spearman correlations. These associations were adjusted for body weight at sacrifice in order to distinguish factual associations from those due to collinearity with weight gain. The heatmap in Fig. 6 illustrates the correlation coefficients of microbiota genera against the eCBome mediators within the ileum. Relative abundances of genera in ileum microbiota, such as *Adlercreutzia*, *Barnesiella*, *Coprobacillus*, *Eubacterium*, and *Parasutterella*, were negatively associated with AEA levels in the small intestine (−0.42 < *r* < −0.37, false-discovery rate [FDR] *q* < 0.1, *n* = 52) (Fig. 6). These associations remained independent of weight even though several of these genera and AEA were concomitantly modified by HFHS feeding. DHEA levels were negatively associated with *Barnesiella*, *Enterococcus*, *Eubacterium*, *Flavonifractor*, and *Intestinimonas* (−0.37 < *r* < −0.30, FDR *q* < 0.1, *n* = 52). Of note, *Barnesiella* and *Intestinimonas* remained associated with DHEA following adjustment for body weight. Such associations were not present with levels of OEA in the ileum, while only *Delftia* was negatively associated with LEA (*r* = −0.32, FDR *q* < 0.1, *n* = 52). Interestingly, all 2‑monoacylglycerols in the ileum were positively associated with ileal *Parasutterella* (0.19 < *r* < 0.35, *n* = 52), and these correlations reached significance for 2-PG, 2-LG, 2-EPG, 2-DHG, and 2-DPG (FDR *q* < 0.1). Similarly, the *Lactobacillus* genus was independently associated with increased 2-DHG levels in the ileum (*r* = 0.42, *q* < 0.01, *n* = 52), which is in marked contrast to its negative association with *N*-acylethanolamines.

We next explored the potential association between gut microbiota composition and circulating eCBome mediators (Fig. S1). The relative abundance of genera in the small intestine microbiota shows only few associations with eCBome mediators in plasma, although *Defluviitalea* and *Flavobacterium*, in the jejunum, and *Adlercreutzia* and *Sporobacter*, in the ileum, were independently associated with circulating 2-AG. Interestingly, the relative abundance of genera in the cecum microbiota also shows some independent associations with circulating levels of eCBome mediators, such as DHEA, 2-OG, and 2-EPG with *Intestinimonas*, *Bacteroides*, and *Sporobacter*, respectively. Taken together, these data suggest that the link between the gut microbiota and the eCBome in metabolic health, by being associated with distant circulating components of this axis, probably extends beyond a local impact.

10.1128/mSystems.00407-19.2 FIG S1 Correlation between intestinal microbiota composition and the plasma endocannabinoidome during HFHS feeding. Heatmaps illustrate correlation coefficients between the relative abundance of jejunal, ileal, and cecal microbiota and the level of endocannabinoidome mediators in the plasma. Boxed cells refer to *q* values of <0.1 of FDR-adjusted Spearman rho correlation; slashed cells refer to *q* values of <0.1 of FDR-adjusted partial Spearman rho correlation accounting for weight at sacrifice (*n* = 49 to 58). Download FIG S1, DOCX file, 0.1 MB. Copyright © 2019 Lacroix et al. 2019 Lacroix et al. This content is distributed under the terms of the Creative Commons Attribution 4.0 International license.

**FIG 7:**
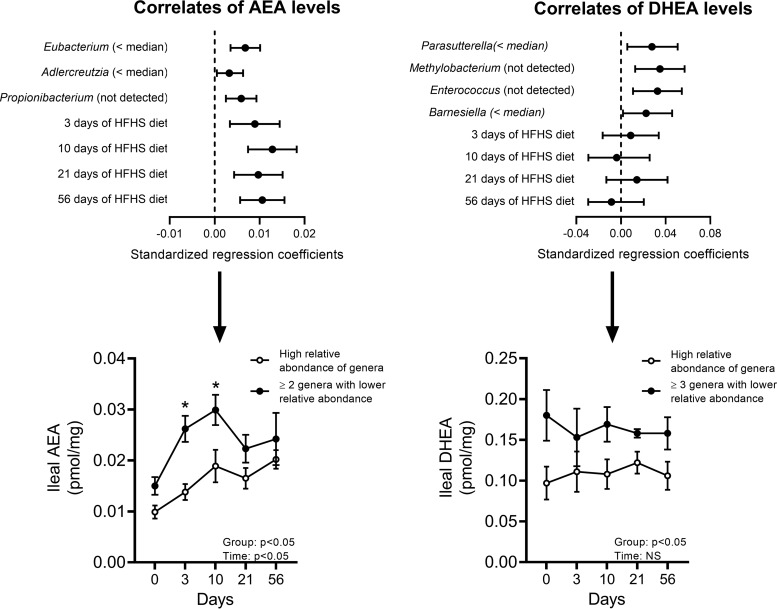
Intestinal microbiota correlates of ileum endocannabinoidome mediator response to the HFHS feeding. (Top) Standardized regression coefficients of intestinal microbiota correlates of AEA and DHEA levels in the ileum. (Bottom) Levels of AEA and DHEA in ileum at each time point according to ileum microbiota profile: nondetectable or low relative abundance versus high relative abundance of genera identified as significant correlates of eCBome mediators. All genera significantly associated with the mediator as well as genera impacted by the HFHS feeding were considered in this analysis. All models also include the HFHS feeding duration. A stepwise selection procedure was used to compute final models. Data are expressed as the mean ± SEM (*n* = 3 to 8 per group and time point). *, *P* < 0.05 for Tukey HSD *post hoc* test between each time point.

In an attempt to integrate changes observed in the gut microbiome-eCBome axis, we then sought to identify the minimal set of ileal microbiota genera that optimally model the levels of each ileal eCBome mediator throughout HFHS feeding (Fig. 7). The obtained regression models revealed that undetectable or low (below median) levels of some bacterial genera were associated with altered ileal concentrations of the eCB AEA and of the PPARα/γ agonist DHEA (18, 19), independently of weight gain. Undetectable or low relative abundances of *Eubacterium*, *Adlercreutzia*, and *Propionibacterium* in the ileum, on the one hand, and time on the HFHS diet, on the other hand, were significant and independent correlates of higher AEA levels at early HFHS feeding time points (Fig. 7). Intriguingly, the stratification of the mice using this model reveals that mice with an ileum microbiota composition characterized by lower relative abundance of at least two of these genera had significantly higher AEA levels at days 3 and 10, i.e., at the onset of glucose intolerance (Fig. 7). Undetectable or low relative abundances of *Parasutterella*, *Methylobacterium*, *Enterococcus*, and *Barnesiella* were significant and independent correlates of elevated ileal DHEA, the levels of which tended to be higher at time zero and lower at the onset of glucose intolerance (Fig. 7). We could not successfully model variations of 2-AG or any other eCBome mediators.

## DISCUSSION

Recent studies have suggested that the host metabolic response to environmental factors, such as the diet, passes through changes in both the gut microbiome and the signaling of eCBome mediators (20). Yet, the interactions between these two fundamental endogenous and exogenous/symbiotic “omes” have only started to emerge (21, 22). This study aimed at identifying such potential interactions during the establishment of diet-induced obesity and some related metabolic complications. Our results point to the existence of a strong association between changes in the relative abundance of certain genera of the intestinal microbiota and the concentrations in the ileum or plasma of certain eCBome mediators during the early onset of HFHS diet-induced glucose intolerance and ensuing obesity and hyperinsulinemia.

We reveal for the first time key segment-specific as well as time-dependent responses to an obesogenic diet in the gut microbiome composition, paralleled by changes in local and circulating concentrations of eCBome mediators. Furthermore, we identified associations of specific bacterial genera (i.e., *Barnesiella*, *Adlercreutzia*, *Parasutterella*, *Propionibacterium*, *Enterococcus*, and *Methylobacterium*) in the small intestine and cecum with local and circulating levels of eCBome mediators (i.e., AEA and DHEA) independently of changes in body weight. Several of these concomitant changes of the gut microbiome and eCBome were observed as early as 3 days following the initiation of the HFHS diet, suggesting that the gut microbiome-eCBome axis contributes to the initial host adaptation to the diet.

Changes in the abundance of some commensal bacteria, on the one hand, and 2-monoacylglycerol and *N*-acylethanolamine levels, on the other hand, have been previously associated with diet-induced obesity. For instance, the present finding of reduced numbers of *Barnesiella* during HFHS in all intestinal segments agrees with the previously reported decrease in the fecal abundance of this genus in high-fat-diet-induced obesity (23). Similarly, decreased numbers of *Parasutterella*, noted here in the jejunum and the ileum, were also associated with obesity (23). *Akkermansia* numbers were reduced here in the small intestine and colon during HFHS feeding, which agrees with the inverse association of this mucin-degrading genus (in particular, the species *A*. *muciniphila*) with obesity, systemic inflammation, and ensuing metabolic disturbances (11). Finally, *Intestinimonas* and *Sphingomonas*, observed here to increase in the ileum and jejunum, respectively, have both been previously associated with obesity (24, 25) and defective leptin signaling in rat models of obesity (26). These, as well as other intestinal microbiome modifications observed here, could result from adaptation to changes in nutrient availability. Nevertheless, we must emphasize that the LFLS and the HFHS diets used here had identical fiber contents and sources, as well as fatty acid compositions, in order to specifically isolate the impact of higher fatty acid/sucrose intake on weight gain/dysmetabolism and gut microbiota.

Our observations of increasing plasma AEA and 2-AG levels with HFHS feeding are also in agreement with a large body of evidence indicating that these mediators are increased in both obese individuals and rodent models of obesity (27, 28). The plasma levels of other 2-monoacylglycerols are instead negatively correlated with body mass index (BMI) in humans (29), which is also in agreement with the decreasing plasma 2-OG and 2-LG levels observed here.

Previous reports may suggest that the associations of eCBome mediators with the relative abundance of some intestinal microbiota genera discussed here are merely the result of similar responses to diet-induced weight gain of these two systems. This might be the case in some instances, although we did show here that many associations persisted when accounting for changes in body weight. Indeed, some commensal bacteria and eCBome mediators may interact with each other independently of the development of obesity, particularly when their concomitant alterations are observed in the same tissue. In particular, the ileal levels of AEA were strikingly modulated in time in a manner opposite those of ileal *Barnesiella*, *Parasutterella*, *Akkermansia*, and *Coprobacillus*, four genera that have been suggested to protect mice from diet-induced dysmetabolism, suggesting that the former effect might be one of the causes of the latter or vice versa. Indeed, previous studies have shown that conditions leading to increased AEA levels are accompanied by decreased predominance of *A. muciniphila* and that probiotic-induced restoration of this beneficial species concomitantly reduces AEA levels (22). Quite intriguing are also the body weight-independent correlations between some genera and *n*-3 polyunsaturated fatty acid-derived eCBome mediators in ileum, which have been proposed to exert anti-inflammatory effects (18). Finally, correlations were also observed between plasma levels of eCBome mediators and the relative abundance of microbiota genera in all three intestinal segments. The significance of such correlations deserves further investigation since the origin of plasma eCBome mediators is not clear. The fact that we did not find the same correlations between plasma mediators and ileal microbiota genera as with ileal mediators might suggest that the small intestine is not a major source of plasma eCBome mediators.

Clearly, the weight-corrected correlations should also not be overinterpreted and should be used instead to direct future mechanistic studies. For example, the effects of the specific manipulation of eCBome mediator levels on gut microbiota composition should be investigated by using genetic knockout or pharmacological inhibition of eCBome metabolic enzymes. Such studies are suggested not only by previous data obtained, for example, with NAPE-PLD null mice (12, 14), but also by the present observation that the diet-induced increase of AEA levels and the decrease of some 2-monoacylglycerols are accompanied, respectively, by corresponding changes in ileal expression of (i) major AEA-degrading enzyme (*Faah*) and (ii) one of the monoacylglycerol-degrading enzymes (*Abhd6*) and the phospholipase C (*Plcb1*) involved in the synthesis of monoacylglycerol precursors, the diacylglycerols. Furthermore, these changes in eCBome mediators should be interpreted in the light of potential concurrent changes in their several molecular targets. For example, we have found here that the HFHS diet causes an immediate decrease in the expression of ileal CB2, suggested to play a protective role against insulin resistance and inflammation, and of the insulin-sensitizing receptor PPARγ. These findings prompt the idea that the diet-induced increases of ileal eCBome mediators may result in preferential binding to CB1 over CB2 or PPARγ, with subsequent increases of intestinal permeability, inflammation, insulin resistance, and other metabolic disturbances. Likewise, the observed decrease of ileal PPARα expression, together with the decrease in the levels of its preferred ligand, OEA, might be indicative of a blunted inhibitory feedback response on fat intake (30).

Indeed, we highlighted how, in the ileum of HFHS diet-fed mice, low abundance of 2 or more metabolically beneficial genera could be associated with local concentrations of AEA and DHEA (and hence of CB1 and PPARα/γ activity) that are themselves associated with the onset of glucose intolerance and local inflammation. These findings suggest that gut microbiota-eCBome interactions of likely metabolic importance should be looked for at the community level, and not only at the single-genus level. They should also open the way to future studies investigating the direct effect on eCBome mediators and targets of gut colonization with species belonging to some of genera found here to correlate with eCBome alterations.

Interestingly, our results suggest time- and intestinal segment-specific bacterial responses to the HFHS diet and thus underline the importance of studying different intestinal segments, at least in animal models where this is more feasible.

### Conclusions.

In summary, the present study reports a time-dependent profiling of the microbiome and the eCBome in different intestinal segments during the development of HFHS diet-induced glucose intolerance and ensuing obesity and hyperinsulinemia. We provided correlative data suggesting that gut microbiome interactions with the eCBome—one of the endogenous signaling systems most involved in metabolic control—may play a crucial role in the development of HFHS diet-induced metabolic disturbances and host-microbiota dysbiosis. Ultimately, the present findings should open the way to mechanistic studies investigating the molecular basis of the gut microbiome-eCBome axis.

## MATERIALS AND METHODS

### Mice.

Sixty 6-week-old C57BL/6J male mice were individually housed in the animal facility of the Institute of Nutrition and Functional Foods. Mice were fed *ad libitum* with a low-fat, low-sucrose purified diet (10% fat and 7% sucrose [LFLS]; Research Diet, NJ, USA) for a 10-day acclimatization period. Mice were then randomly assigned to 6 groups (*n* = 12) fed a high-fat, high-sucrose purified diet (45% fat and 17% sucrose [HFHS]; Research Diet, NJ, USA) for up to 56 days. These dye-free diets harbor comparable fiber contents, and while the HFHS diet had, by design, a higher fatty acid content, the omega-3/omega-6 ratios were comparable (see Table S2 in the supplemental material). Body weight and food intake were monitored twice a week. Six-hour-fasted mice were sacrificed by cardiac puncture to retrieve plasma (1,780 × *g*, 10 min) at either 0 (baseline), 3, 10, 21, or 56 days following HFHS diet initiation, and jejunum and ileum were collected at 10 cm and 2 cm, respectively, from the ileocecal junction. Luminal contents were collected in PBS with gentle scraping. All samples were stored at −80°C until batch analysis. The study was approved by the Laval University animal ethics committee (CPAUL 2017048-1).

10.1128/mSystems.00407-19.5 TABLE S2 Nutritional composition of study diets. Nutrition composition of the low-fat, low-sucrose (LFLS) and the high-fat, high-sucrose (HFHS) diets. Download Table S2, DOCX file, 0.02 MB. Copyright © 2019 Lacroix et al. 2019 Lacroix et al. This content is distributed under the terms of the Creative Commons Attribution 4.0 International license.

### Glucose homeostasis.

Oral glucose tolerance tests (OGTTs) were performed longitudinally in separate groups of mice fed with either an LFLS (*n* = 11) or an HFHS (*n* = 11) diet as previously described (Text S1) (31).

10.1128/mSystems.00407-19.1 TEXT S1 The OGTT procedures and endocannabinoidome mediator quantification protocol. Download Text S1, DOCX file, 0.02 MB. Copyright © 2019 Lacroix et al. 2019 Lacroix et al. This content is distributed under the terms of the Creative Commons Attribution 4.0 International license.

### 16S rRNA gene sequencing.

Intestinal luminal contents were lysed using bead beating (0.1-mm silica beads) before enzymatic digestion with 50 mg of lysozyme and 200 U/μl mutanolysin (37°C, 45 min). Microbial DNA was extracted using the QIAamp DNA Stool minikit (Qiagen, CA, USA), and amplification of the V3-V4 region was performed using the primers 341F (5′-CCTACGGGNGGCWGCAG-3′) and 805R (5′-GACTACHVGGGTATCTAATCC-3′) (Illumina, CA, USA). Libraries were purified using AxyPrep magnetic beads (Axygen Biosciences, CA, USA), and libraries were assessed using DNA 7500 chips (Agilent Technologies, CA, USA) and PicoGreen (Life Technologies, CA, USA). High-throughput sequencing (2- by 300-bp paired end) was performed on a MiSeq platform (Illumina, CA, USA) at the IBIS (Institut de Biologie Intégrative et des Systèmes, Université Laval). Sequences were processed using the DADA2 package (version 1.10.1) (32), and associations with bacterial taxa were obtained using the Ribosomal Database Project reference database release 11. Samples containing less than 5% of all sequences were filtered out, and microbiome abundances were normalized using cumulative sum scaling (CSS; MetagenomeSeq R package) (33). The phyloseq R package (34) was used to calculate Shannon α-diversity indices and to represent relative abundances. Principal-component analysis was performed using the FactoMineR R package (35).

### Endocannabinoidome mediators.

Plasma samples (40 μl) and ileum samples (5 to 10 mg) were extracted, and lipid mediators were measured using high-pressure liquid chromatography–tandem mass spectrometry (HPLC-MS/MS) as previously described (Text S1) (11). The method can differentiate monoacylglycerol isomers at positions 1 and 2, but signals from both isomers of unsaturated fatty acids were summed—and identified as 2-monoacylglycerols—prior to analysis in order to account for their rapid interconversion. However, similar results were obtained when analyzing either 1- and 2-isomers combined or only 2‑monoacylglycerol signals. The following metabolites were quantitated: *N*-palmitoylethanolamine (PEA), *N*-oleoylethanolamine (OEA), *N*-linoleoylethanolamine (LEA), *N*-arachidonoylethanolamine (AEA), *N*-docosahexaenoylethanolamine (DHEA), 2-palmitoyl-glycerol (2-PG), 2-oleoyl-glycerol (2-OG), 2-linoleoyl-glycerol (2-LG), 2-arachidonoyl-glycerol (2-AG), 2-eicosapentaenoyl-glycerol (2-EPG), 2-docosapentaenoyl(*n*-3)-glycerol (2-DPG), and 2-docosahexaenoyl-glycerol (2-DHG).

### Endocannabinoidome enzymes and receptors.

Total RNA from ileum was isolated using the RNeasy minikit according to the manufacturer’s instructions (Qiagen, CA, USA). Reverse transcriptase PCR was performed with a high-capacity cDNA reverse transcription kit (Applied Biosystems, CA, USA). TaqMan assay IDs were as follows: *Tbp* (Mm01277042_m1), *Cnr1* (Mm01212171_s1), *Cnr2* (Mm02620087_s1), *Ppara* (Mm00440939_m1), *Pparg* (Mm00440940_m1), *Abhd6* (Mm00481199_m1), *Plcb1* (Mm00479987_m1), and *Faah* (Mm00515684_m1) (Applied Biosystems, CA, USA). All expression data were normalized by the threshold cycle (2^−ΔΔ^*^CT^*) method using *Tbp* as internal control (36).

### Statistical analyses.

Data are expressed as mean ± SEM (parametric data) or median (Q1-Q3) (nonparametric data). Mixed linear regressions (nlme R package) and two-way analysis of variance (ANOVA) followed by Tukey honestly significant difference (HSD) *post hoc* test or Kruskal-Wallis test followed by Dunn’s multiple comparison test were used to identify significant intestinal segment or time effects and interactions. Time trends of eCBome mediator responses to the HFHS feeding were investigated using the ANOVA linear contrast *post hoc* analysis. Spearman correlations were used to investigate associations between microbiome genera and eCBome mediators. Spearman correlations were adjusted for body weight at sacrifice using the PResidual R package (37). Adjustments for multiple testing were obtained using false-discovery rate (FDR). For linear regression analyses (stats package), all genera associated with eCBome mediators or HFHS diet were considered and expressed as factor (i.e., undetectable/present or below/above the median). The stepwise selection procedure (Backward) using the Akaike information criterion (AIC) was performed to estimate the relative quality of the final model. Results were considered statistically significant at *P* < 0.05 or FDR-adjusted *P* < 0.1. Analyses were performed with R software version 3.4.3.

### Data availability.

Sequencing data for the 16S rRNA sequences were deposited in NCBI GenBank under BioProject ID PRJNA551660.

10.1128/mSystems.00407-19.3 FIG S2 Study design and timeline. Schematic representation of study design, timeline, and interventions performed with each group of mice. Download FIG S2, DOCX file, 1.5 MB. Copyright © 2019 Lacroix et al. 2019 Lacroix et al. This content is distributed under the terms of the Creative Commons Attribution 4.0 International license.
