# Supplementary material for: Rapid and Concomitant Gut Microbiota and Endocannabinoidome Response to Diet-Induced Obesity in Mice
Source: mSystems. 2019 Dec 17;4(6):e00407-19. doi: 10.1128/mSystems.00407-19 (PMC6918026; doi:10.1128/mSystems.00407-19)
Supplement: TABLE S1 [file mSystems.00407-19-st001.docx]

# Table S1:

| **eCBome target** | **eCBome mediators that activate the target** | **Role in energy metabolism of synthetic or endogenous activators of the target** | **Role in the small intestine of synthetic or endogenous activators of the target** | **Role in systemic inflammation of synthetic or endogenous activators of the target** |
| --- | --- | --- | --- | --- |
| **CB1** | AEA, 2-AG [1] | ↑ Adipogenesis and lipogenesis [2,3]  ↑ Glucose intolerance and insulin resistance [4,5]  ↑ Appetite and ↓ satiety [6,7]  ↑ Fatty liver [8,9] | ↓ Motility [10]  ↓ Satiety in response to fat intake [11]  ↑ Paracellular permeability [12,13] | Pro-inflammatory [14] |
| **CB2** | 2-AG > AEA >> DHEA [1,15] | ↑ Insulin sensitivity [16]  ↓Glucose intolerance [17] | ↓ Motility [18] | Anti-inflammatory [19] |
| **TRPV1** | All non-saturated long chain NAEs and 2-MAGs, including AEA and 2-AG [20,21] | ↑ Insulin sensitivity [22]  ↓ Glucose intolerance [23]  ↓ Adipogenesis [24]  ↑ Satiety [25] | ↓ Paracellular permeability [26]  ↑ GLP-1 release [23] | Anti-inflammatory (due to its rapid desensitization) [27] |
| **PPARα** | OEA > PEA > DHEA [28] | ↑ Satiety [29,30]  ↓ Fatty liver [31] | ↑ Satiety in response to fat intake [29]  ↓ Paracellular permeability [26] | Anti-inflammatory [31,32] |
| **PPARγ** | AEA, DHEA [33,34] | ↑ Insulin sensitivity  ↓ Fatty liver [31]  ↑ Adipogenesis/fat remodeling [35] | ↑ Lipid metabolism [36] | Anti-inflammatory [37] |
| **GPR55** | PEA [38] | ↑ Insulin sensitivity [39]  ↑ Insulin release [40] | ↓ Motility [41] | Anti-inflammatory [42] |
| **GPR119** | OEA, LEA, 2-LG, 2-OG [43] | ↑ Glucagon release [44]  ↓ Fatty liver [45] | ↑ GLP-1 release [46,47] |  |

# SUPPLEMENTARY REFERENCES:

1 Sugiura T, Kondo S, Sukagawa A, *et al.* 2-Arachidonoylglycerol: a possible endogenous cannabinoid receptor ligand in brain. *Biochem Biophys Res Commun* 1995;**215**:89–97.

2 Pagano C, Rossato M, Vettor R. Endocannabinoids, Adipose Tissue and Lipid Metabolism. *Journal of Neuroendocrinology* 2008;**20**:124–9. doi:10.1111/j.1365-2826.2008.01690.x

3 Cota D, Marsicano G, Tschöp M, *et al.* The endogenous cannabinoid system affects energy balance via central orexigenic drive and peripheral lipogenesis. *J Clin Invest* 2003;**112**:423–31. doi:10.1172/JCI17725

4 Lipina C, Vaanholt LM, Davidova A, *et al.* CB1 receptor blockade counters age-induced insulin resistance and metabolic dysfunction. *Aging Cell* 2016;**15**:325–35. doi:10.1111/acel.12438

5 Ravinet Trillou C, Delgorge C, Menet C, *et al.* CB1 cannabinoid receptor knockout in mice leads to leanness, resistance to diet-induced obesity and enhanced leptin sensitivity. *Int J Obes Relat Metab Disord* 2004;**28**:640–8. doi:10.1038/sj.ijo.0802583

6 Jamshidi N, Taylor DA. Anandamide administration into the ventromedial hypothalamus stimulates appetite in rats. *Br J Pharmacol* 2001;**134**:1151–4. doi:10.1038/sj.bjp.0704379

7 Kirkham TC, Williams CM, Fezza F, *et al.* Endocannabinoid levels in rat limbic forebrain and hypothalamus in relation to fasting, feeding and satiation: stimulation of eating by 2-arachidonoyl glycerol. *Br J Pharmacol* 2002;**136**:550–7. doi:10.1038/sj.bjp.0704767

8 Osei-Hyiaman D, Liu J, Zhou L, *et al.* Hepatic CB1 receptor is required for development of diet-induced steatosis, dyslipidemia, and insulin and leptin resistance in mice. *J Clin Invest* 2008;**118**:3160–9. doi:10.1172/JCI34827

9 Jourdan T, Djaouti L, Demizieux L, *et al.* CB1 antagonism exerts specific molecular effects on visceral and subcutaneous fat and reverses liver steatosis in diet-induced obese mice. *Diabetes* 2010;**59**:926–34. doi:10.2337/db09-1482

10 Cluny NL, Keenan CM, Duncan M, *et al.* Naphthalen-1-yl-(4-pentyloxynaphthalen-1-yl)methanone (SAB378), a peripherally restricted cannabinoid CB1/CB2 receptor agonist, inhibits gastrointestinal motility but has no effect on experimental colitis in mice. *J Pharmacol Exp Ther* 2010;**334**:973–80. doi:10.1124/jpet.110.169946

11 DiPatrizio NV, Astarita G, Schwartz G, *et al.* Endocannabinoid signal in the gut controls dietary fat intake. *Proc Natl Acad Sci USA* 2011;**108**:12904–8. doi:10.1073/pnas.1104675108

12 Karwad MA, Couch DG, Theophilidou E, *et al.* The role of CB1 in intestinal permeability and inflammation. *FASEB J* 2017;**31**:3267–77. doi:10.1096/fj.201601346R

13 Alhamoruni A, Wright KL, Larvin M, *et al.* Cannabinoids mediate opposing effects on inflammation-induced intestinal permeability. *Br J Pharmacol* 2012;**165**:2598–610. doi:10.1111/j.1476-5381.2011.01589.x

14 Grunewald ZI, Lee S, Kirkland R, *et al.* Cannabinoid receptor type-1 partially mediates metabolic endotoxemia-induced inflammation and insulin resistance. *Physiology & Behavior* 2019;**199**:282–91. doi:10.1016/j.physbeh.2018.11.035

15 Balvers MGJ, Verhoeckx KCM, Plastina P, *et al.* Docosahexaenoic acid and eicosapentaenoic acid are converted by 3T3-L1 adipocytes to N-acyl ethanolamines with anti-inflammatory properties. *Biochim Biophys Acta* 2010;**1801**:1107–14. doi:10.1016/j.bbalip.2010.06.006

16 Zhang X, Gao S, Niu J, *et al.* Cannabinoid 2 Receptor Agonist Improves Systemic Sensitivity to Insulin in High-Fat Diet/Streptozotocin-Induced Diabetic Mice. *Cell Physiol Biochem* 2016;**40**:1175–85. doi:10.1159/000453171

17 Bermudez-Silva FJ, Sanchez-Vera I, Suárez J, *et al.* Role of cannabinoid CB2 receptors in glucose homeostasis in rats. *Eur J Pharmacol* 2007;**565**:207–11. doi:10.1016/j.ejphar.2007.02.066

18 Duncan M, Mouihate A, Mackie K, *et al.* Cannabinoid CB2 receptors in the enteric nervous system modulate gastrointestinal contractility in lipopolysaccharide-treated rats. *Am J Physiol Gastrointest Liver Physiol* 2008;**295**:G78–87. doi:10.1152/ajpgi.90285.2008

19 Parlar A, Arslan SO, Doğan MF, *et al.* The exogenous administration of CB2 specific agonist, GW405833, inhibits inflammation by reducing cytokine production and oxidative stress. *Exp Ther Med* 2018;**16**:4900–8. doi:10.3892/etm.2018.6753

20 Raboune S, Stuart JM, Leishman E, *et al.* Novel endogenous N-acyl amides activate TRPV1-4 receptors, BV-2 microglia, and are regulated in brain in an acute model of inflammation. *Front Cell Neurosci* 2014;**8**:195. doi:10.3389/fncel.2014.00195

21 De Petrocellis L, Nabissi M, Santoni G, *et al.* Actions and Regulation of Ionotropic Cannabinoid Receptors. *Adv Pharmacol* 2017;**80**:249–89. doi:10.1016/bs.apha.2017.04.001

22 Lee E, Jung DY, Kim JH, *et al.* Transient receptor potential vanilloid type-1 channel regulates diet-induced obesity, insulin resistance, and leptin resistance. *FASEB J* 2015;**29**:3182–92. doi:10.1096/fj.14-268300

23 Wang P, Yan Z, Zhong J, *et al.* Transient receptor potential vanilloid 1 activation enhances gut glucagon-like peptide-1 secretion and improves glucose homeostasis. *Diabetes* 2012;**61**:2155–65. doi:10.2337/db11-1503

24 Luo Z, Ma L, Zhao Z, *et al.* TRPV1 activation improves exercise endurance and energy metabolism through PGC-1α upregulation in mice. *Cell Res* 2012;**22**:551–64. doi:10.1038/cr.2011.205

25 Ahern GP. Activation of TRPV1 by the satiety factor oleoylethanolamide. *J Biol Chem* 2003;**278**:30429–34. doi:10.1074/jbc.M305051200

26 Karwad MA, Macpherson T, Wang B, *et al.* Oleoylethanolamine and palmitoylethanolamine modulate intestinal permeability in vitro via TRPV1 and PPARα. *FASEB J* 2017;**31**:469–81. doi:10.1096/fj.201500132

27 Ambrosino P, Soldovieri MV, Russo C, *et al.* Activation and desensitization of TRPV1 channels in sensory neurons by the PPARα agonist palmitoylethanolamide. *Br J Pharmacol* 2013;**168**:1430–44. doi:10.1111/bph.12029

28 Sagar DR, Kendall DA, Chapman V. Inhibition of fatty acid amide hydrolase produces PPAR-alpha-mediated analgesia in a rat model of inflammatory pain. *Br J Pharmacol* 2008;**155**:1297–306. doi:10.1038/bjp.2008.335

29 Schwartz GJ, Fu J, Astarita G, *et al.* The lipid messenger OEA links dietary fat intake to satiety. *Cell Metab* 2008;**8**:281–8. doi:10.1016/j.cmet.2008.08.005

30 Cano C, Pavón J, Serrano A, *et al.* Novel sulfamide analogs of oleoylethanolamide showing in vivo satiety inducing actions and PPARalpha activation. *J Med Chem* 2007;**50**:389–93. doi:10.1021/jm0601102

31 Pawlak M, Lefebvre P, Staels B. Molecular mechanism of PPARα action and its impact on lipid metabolism, inflammation and fibrosis in non-alcoholic fatty liver disease. *J Hepatol* 2015;**62**:720–33. doi:10.1016/j.jhep.2014.10.039

32 Silveira LS, Pimentel GD, Souza CO, *et al.* Effect of an acute moderate-exercise session on metabolic and inflammatory profile of PPAR-α knockout mice. *Cell Biochem Funct* 2017;**35**:510–7. doi:10.1002/cbf.3308

33 Rovito D, Giordano C, Vizza D, *et al.* Omega-3 PUFA ethanolamides DHEA and EPEA induce autophagy through PPARγ activation in MCF-7 breast cancer cells. *Journal of Cellular Physiology* 2013;**228**:1314–22. doi:10.1002/jcp.24288

34 Bouaboula M, Hilairet S, Marchand J, *et al.* Anandamide induced PPARgamma transcriptional activation and 3T3-L1 preadipocyte differentiation. *Eur J Pharmacol* 2005;**517**:174–81. doi:10.1016/j.ejphar.2005.05.032

35 Sharma AM, Staels B. Peroxisome Proliferator-Activated Receptor γ and Adipose Tissue—Understanding Obesity-Related Changes in Regulation of Lipid and Glucose Metabolism. *J Clin Endocrinol Metab* 2007;**92**:386–95. doi:10.1210/jc.2006-1268

36 Duszka K, Oresic M, Le May C, *et al.* PPARγ Modulates Long Chain Fatty Acid Processing in the Intestinal Epithelium. *Int J Mol Sci* 2017;**18**. doi:10.3390/ijms18122559

37 Bassaganya-Riera J, Misyak S, Guri AJ, *et al.* PPAR gamma is highly expressed in F4/80(hi) adipose tissue macrophages and dampens adipose-tissue inflammation. *Cell Immunol* 2009;**258**:138–46. doi:10.1016/j.cellimm.2009.04.003

38 Kramar C, Loureiro M, Renard J, *et al.* Palmitoylethanolamide Modulates GPR55 Receptor Signaling in the Ventral Hippocampus to Regulate Mesolimbic Dopamine Activity, Social Interaction, and Memory Processing. *Cannabis Cannabinoid Res* 2017;**2**:8–20. doi:10.1089/can.2016.0030

39 Lipina C, Walsh SK, Mitchell SE, *et al.* GPR55 deficiency is associated with increased adiposity and impaired insulin signaling in peripheral metabolic tissues. *FASEB J* 2019;**33**:1299–312. doi:10.1096/fj.201800171R

40 Liu B, Song S, Ruz-Maldonado I, *et al.* GPR55-dependent stimulation of insulin secretion from isolated mouse and human islets of Langerhans. *Diabetes Obes Metab* 2016;**18**:1263–73. doi:10.1111/dom.12780

41 Lin X-H, Yuece B, Li Y-Y, *et al.* A novel CB receptor GPR55 and its ligands are involved in regulation of gut movement in rodents. *Neurogastroenterol Motil* 2011;**23**:862-e342. doi:10.1111/j.1365-2982.2011.01742.x

42 Stančić A, Jandl K, Hasenöhrl C, *et al.* The GPR55 antagonist CID16020046 protects against intestinal inflammation. *Neurogastroenterol Motil* 2015;**27**:1432–45. doi:10.1111/nmo.12639

43 Syed SK, Bui HH, Beavers LS, *et al.* Regulation of GPR119 receptor activity with endocannabinoid-like lipids. *Am J Physiol Endocrinol Metab* 2012;**303**:E1469-1478. doi:10.1152/ajpendo.00269.2012

44 Li NX, Brown S, Kowalski T, *et al.* GPR119 Agonism Increases Glucagon Secretion During Insulin-Induced Hypoglycemia. *Diabetes* 2018;**67**:1401–13. doi:10.2337/db18-0031

45 Yang JW, Kim HS, Im JH, *et al.* GPR119: a promising target for nonalcoholic fatty liver disease. *FASEB J* 2016;**30**:324–35. doi:10.1096/fj.15-273771

46 Lan H, Lin HV, Wang CF, *et al.* Agonists at GPR119 mediate secretion of GLP-1 from mouse enteroendocrine cells through glucose-independent pathways. *Br J Pharmacol* 2012;**165**:2799–807. doi:10.1111/j.1476-5381.2011.01754.x

47 Hansen HS, Rosenkilde MM, Holst JJ, *et al.* GPR119 as a fat sensor. *Trends Pharmacol Sci* 2012;**33**:374–81. doi:10.1016/j.tips.2012.03.014

48 Everard A, Belzer C, Geurts L, *et al.* Cross-talk between Akkermansia muciniphila and intestinal epithelium controls diet-induced obesity. *PNAS* 2013;**110**:9066–71. doi:10.1073/pnas.1219451110
