# Supplementary figures and images for: Rapid and Concomitant Gut Microbiota and Endocannabinoidome Response to Diet-Induced Obesity in Mice
Source: mSystems. 2019 Dec 17;4(6):e00407-19. doi: 10.1128/mSystems.00407-19 (PMC6918026; doi:10.1128/mSystems.00407-19)

#
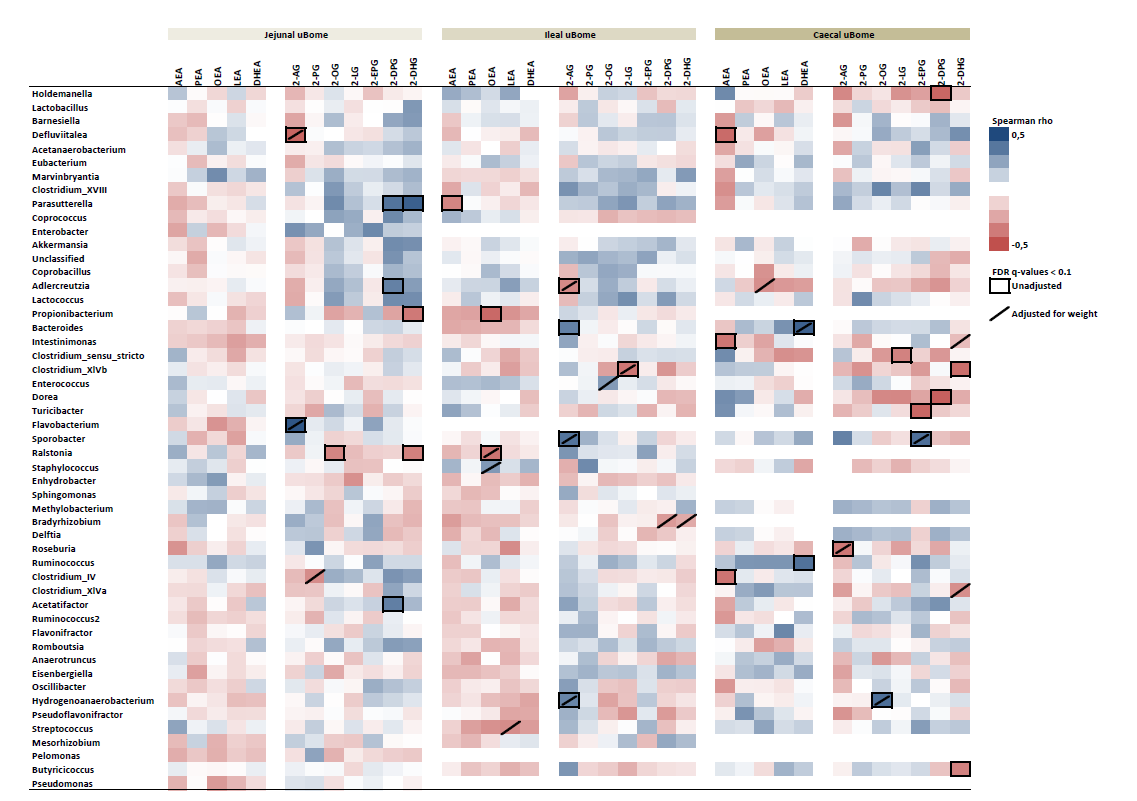
Figure S1

Supplement: FIG S1 [file mSystems.00407-19-sf001.docx]

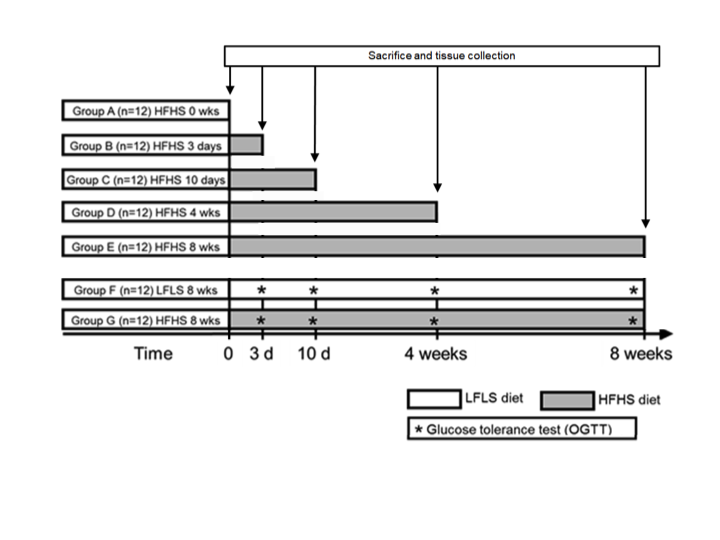

Supplement: FIG S2 [file mSystems.00407-19-sf002.docx]
