# Supplementary material for: Rapid and Concomitant Gut Microbiota and Endocannabinoidome Response to Diet-Induced Obesity in Mice
Source: mSystems. 2019 Dec 17;4(6):e00407-19. doi: 10.1128/mSystems.00407-19 (PMC6918026; doi:10.1128/mSystems.00407-19)
Supplement: TABLE S2 [file mSystems.00407-19-st002.docx]

|  | LFLS diet (D12450) | HFHS diet (D12492) |
| --- | --- | --- |
| Energy (kcal) from protein (%) | 20 | 20 |
| Energy (kcal) from carbohydrate (%) | 70 | 35 |
| Energy (kcal) from fat (%) | 10 | 45 |
|  |  |  |
| Sucrose (%) | 7 | 17 |
|  |  |  |
| SFA (g/100g) | 1,1 | 8,5 |
| MUFA (g/100g) | 1,4 | 10 |
| PUFA (g/100g) | 1,6 | 3,9 |
| n-3 linolenic acid (g/100g) | 0,2 | 0,4 |
| n-6 linoleic acid (g/100g) | 1,4 | 3,5 |
| Total fat (g/100g) | 4,2 | 22,4 |
|  |  |  |
| Cholesterol (g/100g) | 1,8 | 19,6 |
|  |  |  |
| Ratio n-6/n-3 | 7,3 | 8,9 |

# Table S2
