# Supplementary material for: Rapid and Concomitant Gut Microbiota and Endocannabinoidome Response to Diet-Induced Obesity in Mice
Source: mSystems. 2019 Dec 17;4(6):e00407-19. doi: 10.1128/mSystems.00407-19 (PMC6918026; doi:10.1128/mSystems.00407-19)
Supplement: TEXT S1 [file mSystems.00407-19-s0001.docx]

# SUPPLEMENTARY METHODS:

## Glucose homeostasis

Oral glucose tolerance tests (OGTT) were performed longitudinally in separate groups of mice ~~of mice~~ fed with either a LFLS (n = 11) or a HFHS diet (n = 11) as previously described [16]. Briefly, 6-hour fasted mice were given 10% dextrose (1 g/kg of body weight, 10ul/g) by gavage and blood was collected at the baseline, 15, 30, 60, and 120 min via the saphen vein without anaesthetics. Glycemia was measured using a OneTouch Verio Flex glucometer. Blood drawn in EDTA coated tubes were centrifuged (1780 g, 10 min) to collect plasma. Snap frozen plasma were stored at -80°C until plasma batch analysis of insulin levels by ELISA (Alpco, NH, USA). Glucose and insulin response were expressed as the incremental area under the curve (iAUC).

## Endocannabinoidome mediators

Plasma samples (40 µl) were brought to 500 µl in 50 mM Tris (pH 7) then mixed with 500 µl methanol containing 0.01% acetic acid and 5 ng of deuterated standards as previously described (Supplementary Methods) [11]. Ileum samples (5 – 10 mg) were crushed with a disposable tissue grinder, harvested with 500 µl PBS then immediately denatured with 500 µl methanol containing 0.1% acetic acid and 5 ng of deuterated standards. Lipids were extracted 3 times by adding 1 ml chloroform, vortexing for 1 minute and centrifuging (3500 g, 10 min). Pooled organic phases were evaporated under nitrogen and resuspended in 50 µl of our mobile phase (50% B). Samples were injected (40 μl) onto an HPLC column (Kinetex C8, 150×2.1 mm, 2.6 μm, Phenomenex) and eluted at a flow rate of 400 μl/min using a discontinuous gradient solvent A (1 mM ammonium acetate + 0,05% acetic acid) and solvent B (acetonitrile/water; 95/5 + 1 mM ammonium acetate + 0.05 acetic acid) as described before. The HPLC system was interfaced with the electrospray source of a Shimadzu 8050 triple quadrupole mass spectrometer and mass spectrometric analysis was done in the positive ion mode using multiple reaction monitoring. The method can differentiate monoacylglycerol isomers at positions 1 and 2 but signals from both isomers of unsaturated fatty acids were summed – and identified as 2- monoacylglycerols – prior to analysis in order to account for their rapid interconversion. However, similar results were obtained when analyzing either 1- and 2- isomers combined or using only 2‑monoacylglycerol signals.
